# Residual bioefficacy of attractive targeted sugar bait stations targeting malaria vectors during seasonal deployment in Western Province of Zambia

**DOI:** 10.1186/s12936-024-04990-3

**Published:** 2024-05-29

**Authors:** Gift Mwaanga, Jacob Ford, Joshua Yukich, Benjamin Chanda, Ruth A. Ashton, Javan Chanda, Buster Munsanje, Emliny Muntanga, Malon Mulota, Christine Simuyandi, Boyd Mulala, Limonty Simubali, Kochelani Saili, Edgar Simulundu, John Miller, Busiku Hamainza, Erica Orange, Joseph Wagman, Monicah M. Mburu, Angela F. Harris, Julian Entwistle, Megan Littrell

**Affiliations:** 1 Macha Research Trust, Choma, Zambia; 2 Center for Applied Malaria Research and Evaluation, Department of Tropical Medicine, Tulane University School of Public Health and Tropical Medicine, New Orleans, USA; 3 PATH, Lusaka, Zambia; 4 PATH, Seattle, USA; 5 PATH, Washington DC, USA; 6 IVCC, Liverpool, UK; 7 National Malaria Elimination Centre, Lusaka, Zambia

## Abstract

**Background:**

The primary vector control interventions in Zambia are long-lasting insecticidal nets and indoor residual spraying. Challenges with these interventions include insecticide resistance and the outdoor biting and resting behaviours of many *Anopheles* mosquitoes. Therefore, new vector control tools targeting additional mosquito behaviours are needed to interrupt transmission. Attractive targeted sugar bait (ATSB) stations, which exploit the sugar feeding behaviours of mosquitoes, may help in this role. This study evaluated the residual laboratory bioefficacy of Westham prototype ATSB® Sarabi v.1.2.1 Bait Station (Westham Ltd., Hod-Hasharon, Israel) in killing malaria vectors in Western Province, Zambia, during the first year of a large cluster randomized phase-III trial (Clinical Trials.gov Identifier: NCT04800055).

**Methods:**

This was a repeat cross-sectional study conducted within three districts, Nkeyema, Kaoma, and Luampa, in Western Province, Zambia. The study was conducted in 12 intervention clusters among the 70 trial clusters (35 interventions, 35 controls) between December 2021 and June 2022. Twelve undamaged bait stations installed on the outer walls of households were collected monthly (one per cluster per month) for bioassays utilizing adult female and male *Anopheles gambiae *sensu stricto (Kisumu strain) mosquitoes from a laboratory colony.

**Results:**

A total of 84 field-deployed ATSB stations were collected, and 71 ultimately met the study inclusion criteria for remaining in good condition. Field-deployed stations that remained in good condition (intact, non-depleted of bait, and free of dirt as well as mold) retained high levels of bioefficacy (mean induced mortality of 95.3% in males, 71.3% in females, 83.9% combined total) over seven months in the field but did induce lower mortality rates than non-deployed ATSB stations (mean induced mortality of 96.4% in males, 87.0% in females, 91.4% combined total). There was relatively little variation in corrected mortality rates between monthly rounds for those ATSB stations that had been deployed to the field.

**Conclusion:**

While field-deployed ATSB stations induced lower mortality rates than non-deployed ATSB stations, these stations nonetheless retained relatively high and stable levels of bioefficacy across the 7-month malaria transmission season. While overall mean mosquito mortality rates exceeded 80%, mean mortality rates for females were 24 percentage points lower than among males and these differences merit attention and further evaluation in future studies. The duration of deployment was not associated with lower bioefficacy. Westham prototype ATSB stations can still retain bioefficacy even after deployment in the field for 7 months, provided they do not meet predetermined criteria for replacement.

**Supplementary Information:**

The online version contains supplementary material available at 10.1186/s12936-024-04990-3.

## Background

Malaria remains a leading cause of morbidity and mortality in endemic regions of sub-Saharan Africa [1]. The most common methods of malaria vector control are indoor-based and include the use of long-lasting insecticidal nets (LLINs) and indoor residual spraying (IRS). These measures have substantially contributed to the observed global reduction in malaria burden since 2000 [2–6].

However, the effectiveness of indoor-based malaria vector control measures is threatened. This is due to (a) insecticide resistance, which is expanding and intensifying in vector populations across Africa [7–14], and (b) a shift toward a higher abundance of outdoor biting and resting behaviours, which is recognized as a considerable potential threat for the future. As indoor interventions successfully reduce the malaria vectors responsible for indoor transmission, the importance of addressing outdoor-biting vectors with appropriate tools is increasingly critical to sustain gains in malaria control [15–18]. Progress toward global malaria burden reduction goals has slowed, and key targets of the WHO’s Global Malaria Programme (GMP) technical strategy may be missed partially due to these threats to indoor vector control tools [19–21]. Therefore, there is a need to develop, evaluate and scale-up complementary malaria vector control tools that target outdoor and other residual transmission. One promising new approach is the use of attractive targeted sugar bait (ATSB) stations. This intervention exploits the natural sugar feeding behaviours of mosquitoes [22–25], unlike the blood-feeding and resting behaviours exploited by LLINs and IRS, respectively [26, 27]. Attractive targeted sugar baits use a combination of attractive sugar-based bait and an ingestion toxicant in an attract-and-kill approach. The advantages of using ATSB stations include that they target a novel part of the mosquito life cycle and can be deployed outside the home. Additionally, ATSB stations use ingestion toxicants rather than contact insecticides, with many potential active ingredient options available for use [22–24].

The ATSB^®^ Sarabi v.1.2.1 Bait Station (Westham Ltd., Hod-Hasharon, Israel) was designed to attract and kill malaria vectors. A proof-of-concept entomological field trial of an earlier prototype Westham ATSB Bait Stations was completed in Mali, containing the active ingredient dinotefuran 0.11% (w/w), 1% (w/w) BaitStab (a product containing antibacterial and antifungal additives), with natural sugars (~ 75 Degrees Brix). The proof-of-concept trial against *Anopheles gambiae *sensu lato (*s.l*.) in the tropical savannah of the Kayes region in Mali demonstrated high ATSB feeding rates and significant reductions in *An. gambiae s.l.* density, biting rates, and overall entomological inoculation rate (EIR) [22].

Following these favourable results in Mali, the ATSB research team in Zambia in collaboration with the National Malaria Elimination Programme (NMEP) under the Zambian Ministry of Health (MoH) was engaged to conduct a series of entomological studies to investigate the potential for the ATSB® Sarabi Station to control malaria vectors at study sites in the more temperate, rainier region of Western Province, Zambia where resistance to multiple pyrethroids is prevalent in both *Anopheles funestus* and *An. gambiae* but both vector populations are susceptible to pirimiphos-methyl and dinotefuran (Wagman et al., pers. commun.). Following a series of cage-mortality assays and semi-field studies in Zambia, the ATSB research team designed the ATSB bioassay study to evaluate the bait stations’ residual efficacy in killing mosquitoes throughout their deployment in communities. This study was completed as part of a Phase III cluster-randomized controlled trial designed to determine if these ATSB can be deployed to reduce malaria incidence and prevalence [28] in the context of universal deployment of other indoor-based malaria vector control interventions (LLINs and IRS) (ClinicalTrials.gov Identifier: NCT04800055).

### Objective

The primary objective of the study was to evaluate the induced mortality in bioassays of ATSB stations deployed in communities during a randomized trial for killing mosquitoes throughout a 7-month deployment period.

## Methods

### Study sites

The study was conducted in 12 clusters in the ATSB Phase III trial. Clusters selected included all ten intervention-arm clusters where entomological surveillance was conducted, plus two additional clusters chosen to ensure geographic representation across the whole trial site [28]. The clusters were within three districts: Nkeyema, Kaoma and Luampa districts in Western Province, Zambia. Malaria transmission in Western Province is known to be seasonal, typically characterized by peak transmission from January to May, relating to the annual rainy season, which typically lasts from November to March. This study was conducted for seven months during the rainy season to the middle of the cold season (from December 2021 to June 2022).

### Study design

This was a repeat cross-sectional study design incorporated in the ATSB main trial (ClinicalTrials.gov Identifier: NCT04800055). Briefly, an enumeration activity was used to identify 70 clusters of approximately 175 households each. Restricted randomization was used to assign 35 clusters to the intervention arm (ATSB plus IRS/LLIN) and 35 clusters to the control arm (IRS/LLIN alone). Clusters used a ‘fried egg’ design whereby the intervention was deployed in the cluster ‘core’ plus a buffer area extending 600 m beyond, while sampling for outcome ascertainment for the epidemiological outcomes was only conducted in the core areas. ATSB stations were installed on eligible structures found within the entire cluster, including the buffer and core zones. Each eligible structure received two ATSB stations during an installation campaign in the first two weeks of November 2021. The ATSB stations were monitored throughout the trial period and replaced by ATSB monitors if they met predefined criteria of damage, including evidence of defined levels of mold, leakage, holes/tears, dirt, or depletion of the bait.

**Fig. 1:**
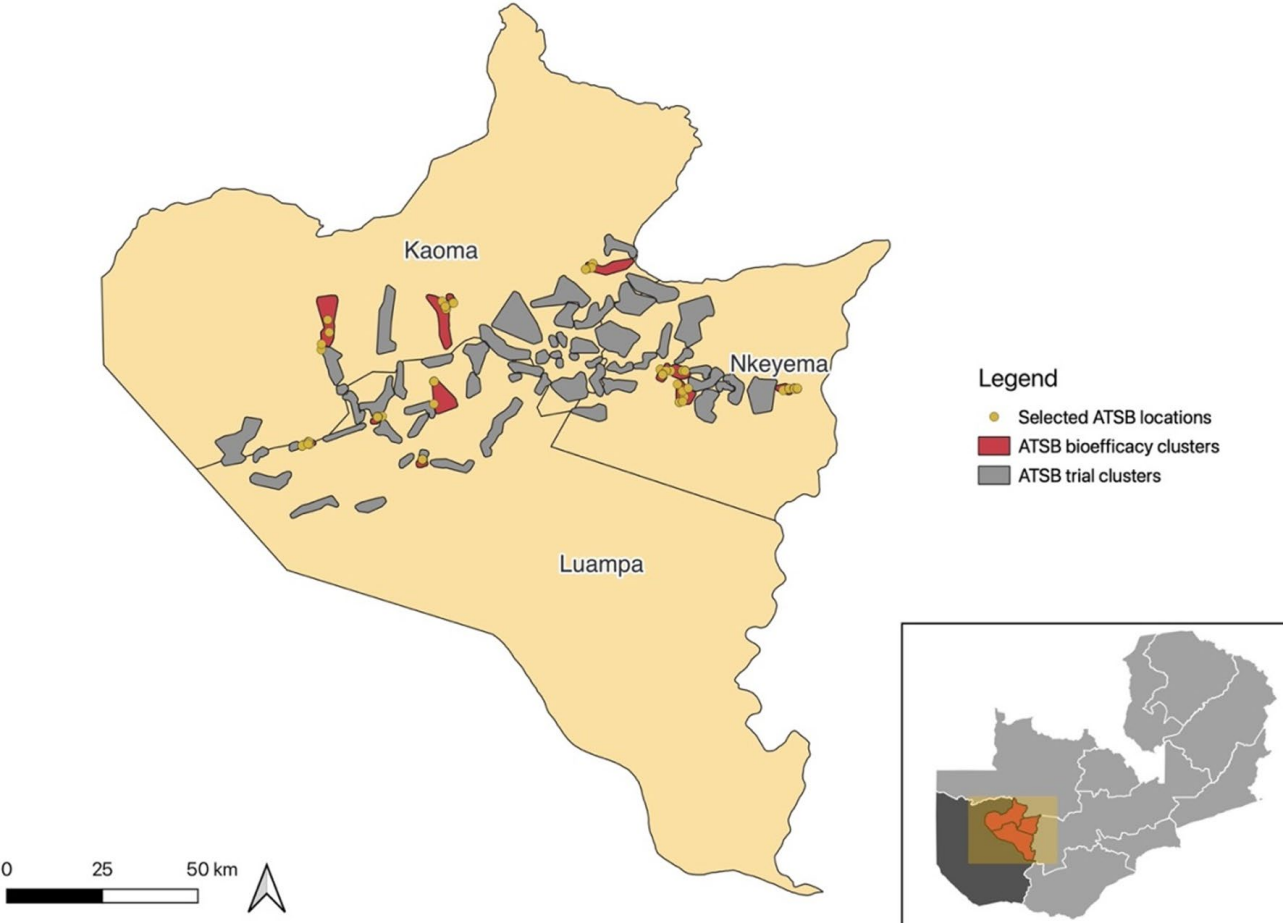
Map of study area, highlighting the three study districts in Western Province, Zambia, the 12 trial clusters participating in bioefficacy assessment, and location of ATSBs selected for bioefficacy testing

Only ATSB stations installed during the initial November 2021 campaign in 12 of the 35 intervention clusters that were selected for this sub-study were eligible for this residual bioefficacy study (Fig. 1). ATSB stations that were installed as replacements for damaged or missing stations throughout the study period were not eligible for this study. Selected clusters included 10 clusters previously selected for mosquito sampling, plus two additional clusters chosen to ensure geographic representation across the whole trial site. Once per month, one ATSB not meeting replacement criteria was removed from a structure in each cluster (for a total of 12 ATSB stations per month) and transferred to the laboratory at Macha Research Trust for bioefficacy testing. The bioefficacy evaluations were conducted monthly for seven months, beginning in December 2021 after ATSB stations had been deployed for one month.

### ATSB stations

**Fig. 2:**
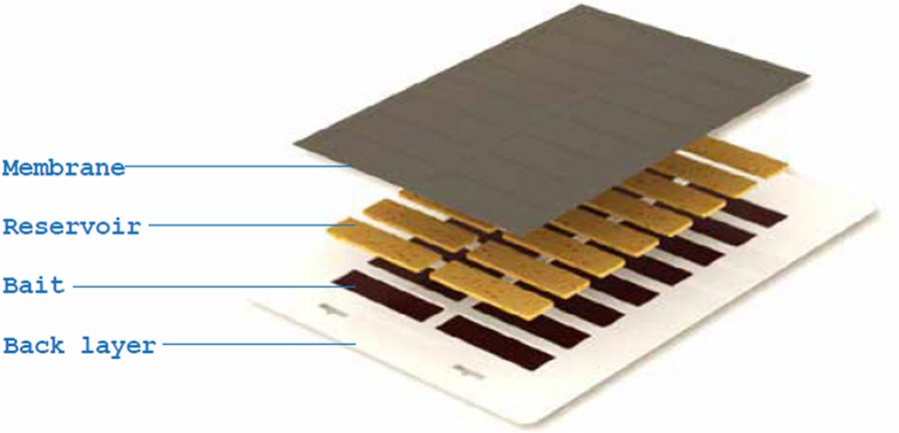
An ATSB station The ATSB station is made of a plastic layer, 16 cells that serve as the reservoir for the bait, and a protective membrane that covers the bait but allows mosquitoes to feed

The product evaluated was the Sarabi version 1.2.1 prototype ATSB station (Fig. 2). All the bait stations had unique preprinted QR codes. Digital photos, taken using mobile devices, included a photograph of the location of each ATSB prior to removal for the bioefficacy study. Photos were visually reviewed by a single investigator, and characteristics of the structure on which ATSB stations were hung were extracted and recorded for each station sampled, including wall type (mud, concrete block, or plaster covered block), roof material (thatch or tin) and presence or absence of roof overhang.

### ATSB installation

**Fig. 3:**
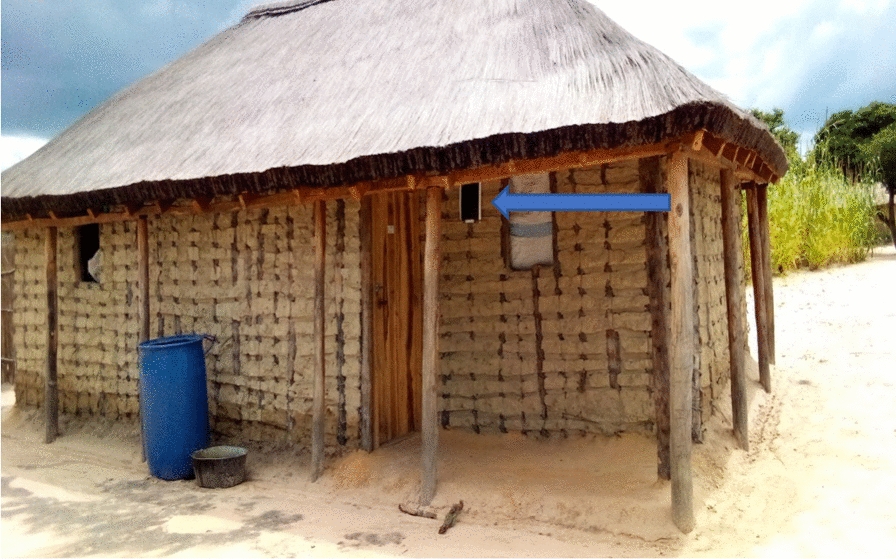
Deployed ATSB bait station on residential structure

The installation of ATSB stations on household structures in the study areas was based on standard eligibility criteria (Fig. 3). Structures that were eligible to receive the ATSB stations were defined as those with (1) a complete roof, (2) walls at least 1 m high, and (3) at least 3 complete walls. Nonresidential buildings (*e.g.,* shops, schools, churches, tobacco drying sheds), kitchens that are not used for sleeping, animal kraals, toilets, bathing shelters, and drying racks were not eligible for ATSB installation. Two ATSB stations were hung using bamboo sticks, nails, and wires on each eligible structure. The bait stations were hung approximately 1.6 – 1.8 m above the ground (where possible) on opposite exterior walls, prioritizing protected locations under an eave or roof overhang.

### Bioefficacy assessment

#### Procedure

Each month, a list of eligible ATSB stations was compiled for each of the twelve selected bioefficacy assessment clusters, where eligibility was defined by having been installed in the first two weeks of November and having no record of removal during routine ATSB monitoring (i.e., monthly inspection of ATSB stations by the ATSB monitors). Since monthly inspection of ATSB station conditions and replacement of those that had incurred damage, started leaking or became dirty or moldy was conducted, some stations that had been deployed at the start of the trial had already been removed and replaced prior to each bioefficacy collection, and those stations that had been removed and their replacements were not considered eligible for selection in this study. Using a cluster map and the list of eligible ATSB stations, two study team members (GM and BC) alongside cluster ATSB monitors visited each cluster core and buffer area monthly to locate, capture data, and remove one eligible ATSB. The first ATSB station encountered in each cluster that did not meet the damage or replacement criteria, but whose barcode was included on the list of eligible stations was removed for bioefficacy testing. Removed ATSB stations were immediately replaced with a new ATSB. The ATSB team explained to the household head that the reason for removing a bait station was to check its operational effectiveness in killing mosquitoes in the communities during the hanging period.

#### Inclusion criteria

1. Bait stations located within the buffer zones and core areas of 12 participating clusters.
2. Bait stations originally installed in November 2021 (have not been removed or replaced since the original installation period).
3. Undamaged (does not meet the criteria for replacement): not leaking, no holes or tears, no mold spots larger than the rubber of a pencil, fewer than 8 depleted cells (i.e., containing low levels of bait), and fewer than 8 cells covered with dirt.

#### Exclusion criteria

1. ATSB stations with 1 or more cells completely torn open, leaking off of the black membrane, depletion (8 or more cells), or dirt (8 or more cells).
2. ATSB stations with mold spots exceeding the size of the rubber end of a pencil.
3. ATSB stations deployed after the November 2021 installation period.

#### Safety measures on handling the ATSB bait stations

The personnel who were handling ATSB stations from the field site were provided with gloves for proper handling and removal of ATSB stations. Personal protective equipment, including laboratory coats and masks, was used during bioassays to avoid the inhalation of mold spores and to mitigate potential hazards associated with handling bait stations.

#### Data capture during bait station removal

Data capture was completed in Commcare (Diamgi Inc., Cambridge MA, USA) using Android smartphones at the time of removal of ATSB stations selected for bioefficacy. Data were recorded by a study team member familiar with the overall ATSB trial’s standardized ATSB damage criteria. This included the collection of the GPS coordinates of the identified structure at the entrance door and ATSB barcode. Data forms also included the capturing of the following characteristics: close-up photograph of ATSB, whether the ATSB had any holes and if any cells were completely torn open, if the ATSB was leaking (liquid dripping from the black surface onto white border or other surfaces), if 0 or 1–7 or 8 + cells were depleted, if 0 or 1–7 or 8 + cells were dirty, presence of any mold and if any mold spots were greater than the size of the rubber end of a pencil, a photo of ATSB’s position on the structure, and the cardinal direction the ATSB was facing while installed (e.g., north, south, etc.).

#### Packaging, storage and transportation of ATSB stations

The removed ATSB stations were labeled with permanent markers as follows: the cluster number, month (i.e., M1 for month one), and date of removal. The bait stations were placed in a predesigned slot in a lockable wooden box for transport to the Macha Research Trust (MRT) for bioefficacy assessment. The wooden box had 12 removable wooden slots to which the ATSB stations were attached, so they were held vertically when transported. Bait stations were transported to MRT within a 4-day ± 1-day span (2–3 days collection and 1 day transit from Kaoma, Western Province to MRT) in the second week of every calendar month from December 2021 to June 2022. The ATSB stations were stored in a transport box awaiting transportation and prior to the assays at MRT.

#### Mortality assessment of the bait stations

Prior to the assessment, the ATSB stations were kept in a transport box within the laboratory for two days prior to the bioassays. ATSB stations were not cleaned or wiped to avoid altering the condition they were in when recovered from the field. The ATSB stations were tested using a laboratory colony of insecticide-susceptible *An. gambiae *sensu stricto (*s.s*.) (Kisumu strain) mosquitoes. Prior to the assessment, female and male mosquitoes of known age (3–5 days old) were reared under controlled conditions (hum; 80 ± 10%, temp; 27 ± 2 °C) in an insectary at Macha Research Trust. The conditions for the second insectary where the experiments were carried out were maintained within the same range.

Female and male mosquitoes were selected, placed in separate cages, and starved (no sugar) for 24 and 12 h, respectively. Water-soaked cotton wool was provided in every cage. After the starvation period, mosquitoes were placed into release cups (50 in each cup or 25 in each cup if using small release cups). The mosquitoes were allowed to stay in cups for a minimum of one hour before releasing them into cages to allow them to stabilize from any stress that may have been incurred during the selection process.

**Fig. 4:**
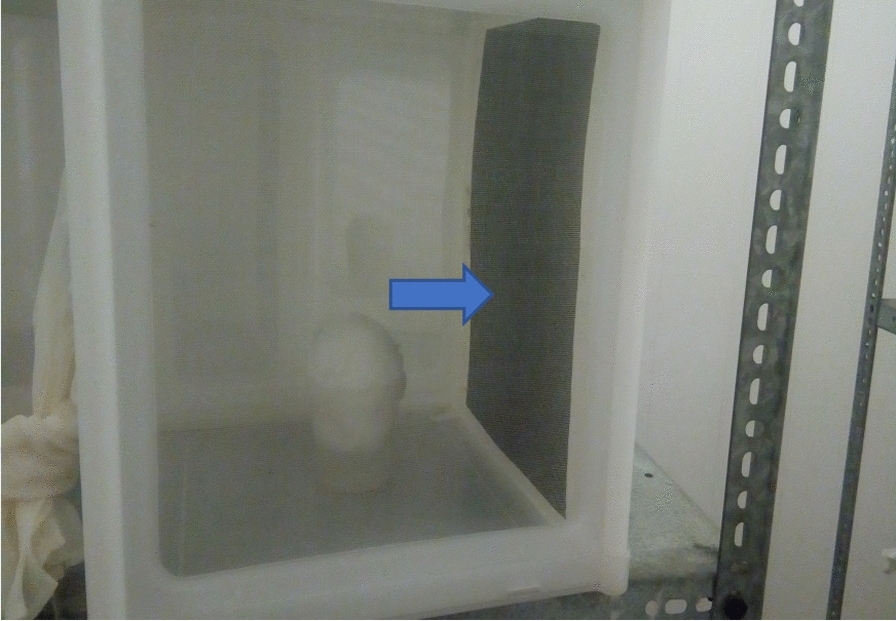
Mounted ATSB station in a cage in portrait position

A total of 84 field-deployed bait stations were collected, 12 every month for seven months. Out of the total collected, 71 were evaluated. Each bait station was mounted inside one of the walls of a separate 30 cm × 30 cm cage in portrait orientation (Fig. 4). Single control cages with water only and with both water and 77% sugar solutions were also constructed to assess control water only and sugar-fed mortality.

Cohorts of fifty starved male and fifty starved female mosquitoes were released into each testing cage and each of two control cages. To ensure that all released mosquitoes were in good condition, mosquitoes were allowed to fly out of the release cups by gently tapping on the side of the cups and removing the cups with any mosquitoes that did not leave the release cup. All cages were provided with cotton wool soaked in water. The cages were all covered with wet towels on the top surface to increase the humidity levels. The temperature/humidity was noted at the start and end of the experiments.

#### Data collection, management and analysis

In each of the eight assay rounds (seven from field-collected ATSB stations and one from the new non-deployed stations), mosquitoes were exposed for 48 h to the two negative controls and to recently removed ATSB stations that met the inclusion criteria (up to 12 ATSB stations/month). In addition, 12 new, non-deployed ATSB stations were bio-assayed in month 0 as a positive control. The dead mosquitoes were counted and removed at 24 h for each treatment, including the control cages, and recorded in a standardized Excel worksheet. The remaining mosquitoes in each of the cages were left for an additional 24 h. At the end of 48 h, the numbers of dead and living mosquitoes in all treatment and control cages were recorded in a standardized Excel worksheet. All data analyses and manipulation were conducted with R (v. 4.1.3) and R Studio (v. 2022.07.1, build 554) [29, 30].

Bioassay mortality was calculated by summing the number of mosquitoes that had died within 24 and 48 h and dividing by the total number of mosquitoes released to feed on the ATSB in each assay. This proportion was then corrected for starvation and natural mortality rates from active sugar or water controls for each round of assays using Abbott’s formula:

$$\frac{\left(\% observed mortality-\% control mortality\right)}{(1-\% control mortality)}$$

The corrected natural mortality proportions were used for subsequent analyses. Bivariate and multivariable linear regression analyses were conducted using the corrected natural mortality (sugar control) proportion as the outcome. Predictor variables examined were condition (new/field deployed), holes, leaks, mold, depletion, dirtiness, wall material and roof material. Predictor variables were used to determine the potential factors that may influence the bioefficacy of field-deployed ATSB. Unadjusted estimates are presented as well as adjusted estimates that account for trial round as both a continuous and categorical (independent) variable.

## Results

### Conditions of the ATSB as recorded by the study team during their collection and from an independent review of photographs taken at the time of collection

Of the 84 ATSB stations collected from the field, 71 met the inclusion criteria upon examination (see Table 1). From the ATSB collections, only two out of the seven months (M2 and M5) had all 12 stations eligible. In most months (M1, M4, M6 and M7), 11 of the 12 stations collected were eligible. There were only three stations eligible during the month three collections. Twelve new, non-deployed ATSB stations were also included as positive controls (total ATSB used in analysis (n = 83); the twelve non-deployed ATSB stations were not included in the analysis of condition. Nearly all eligible selected field-deployed ATSB stations (n = 71) had no holes or leaks and were not considered to be depleted of bait at the time of collection, consistent with the targeted selection criteria for inclusion of field-deployed ATSB stations in the study. The majority of the eligible field-collected ATSB stations (n = 58, 81.7%) had some mold, while 39.4% of collected ATSB stations were considered dirty at the time of collection but below the thresholds for replacement. Most eligible ATSB stations (91.5%) were hung under a thatch roof, and all appeared to be protected by a roofline extending well beyond the outside wall of the house on which they were hung. Bivariate analysis of condition in this limited sample found no statistically significant associations of corrected cumulative 48-h mortality with condition except for being deployed ($\beta =-0.08$, *p* = 0.027) and being dirty ($\beta =-0.06$, *p* = 0.019).

**Table 1:** Shows the conditions of the ATSB as recorded by the study team during their collection as well as the hanging conditions of these ATSB during deployment

Group	Characteristic	N	N = 83^1^
All ATSB stations	Condition	83	
	New		12 (14.5%)
	Field-deployed		71 (85.5%)
Field-deployed ATSB stations	Study month collected	71	
	1		11 (15.5%)
	2		12 (16.9%)
	3		3 (4.2%)
	4		11 (15.5%)
	5		12 (16.9%)
	6		11 (15.5%)
	7		11 (15.5%)
	Holes	71	
	No holes		69 (97.2%)
	Holes		2 (2.8%)
	Leaks	71	
	No leaks		71 (100.0%)
	Leaks		0 (0.0%)
	Mold	71	
	No mold		13 (18.3%)
	Mold		58 (81.7%)
	Depletion	71	
	Full		68 (95.8%)
	Depleted		3 (4.2%)
	Dirtiness	71	
	Not dirty		43 (60.6%)
	Dirty		28 (39.4%)
	Wall material	71	
	Mud		23 (32.4%)
	Brick		33 (46.5%)
	Plaster		15 (21.1%)
	Roof material	71	
	Thatch		65 (91.5%)
	Tin		6 (8.5%)

^1^n (%)

### Natural control mortality

24-h sugar-fed female mortality remained below 5% except for round five, where mortality was 11.43% (Table S1).

### Corrected mortality (natural or starvation) over the number of months for all ATSB in the study

Mortality among male mosquitoes corrected for natural mortality ranged from 92.7% to 97.1%, with mean mortality 95.3% among all assessment with field-deployed ATSB stations (Table 2). Female mortality rates were generally lower, with corrected mean monthly mortality ranging from 61.6% to 72.6% across the seven months, and overall female mortality when exposed to field-deployed ATSB stations was 71.3% (Table 2). The total corrected mortality rate (combining male and female data) for field-deployed ATSB stations was 83.9%. Unadjusted mortality rates are reported in Table 1S.

**Table 2:** Summary of 48-h mosquito mortality by sex and per trial, corrected for natural mortality

	Males tested		Male mortality		% male mortality		Females tested		Female mortality		% female mortality		Total tested		Total mortality		% total mortality	
Trial	Mean	SD	Mean	SD	Mean	SD	Mean	SD	Mean	SD	Mean	SD	Mean	SD	Mean	SD	Mean	SD
0	43.0	4.2	41.6	4.9	96.4	6.7	50.1	5.8	44.2	8.3	87.0	9.4	93.1	7.7	85.8	10.6	91.4	6.2
1	52.3	7.6	50.9	7.4	97.1	3.1	47.8	10.5	34.6	9.6	72.6	18.2	100.1	7.5	85.6	10.2	84.6	10.4
2	54.2	4.7	51.1	3.8	94.6	6.6	49.0	3.8	35.3	11.3	68.4	20.4	103.2	4.8	86.4	12.9	82.8	11.4
3	63.3	17.0	59.7	13.6	94.4	5.0	56.7	4.2	44.0	6.9	73.8	10.3	120.0	21.0	103.7	20.1	84.4	4.6
4	53.9	5.2	52.6	4.0	97.8	6.8	52.6	7.7	47.1	4.9	90.3	7.5	106.5	7.5	99.7	5.8	93.6	4.6
5	48.5	7.8	44.8	7.3	92.7	15.2	28.6	5.9	20.0	4.7	67.4	17.5	77.1	10.9	64.8	10.1	82.9	13.8
6	55.4	6.6	53.2	6.8	95.7	5.0	45.6	8.8	30.2	10.6	61.6	20.8	100.9	9.7	83.4	10.8	81.2	10.4
7	63.8	11.7	62.0	11.2	94.5	6.2	65.6	9.4	44.6	13.8	65.0	18.6	129.4	17.2	106.6	18.4	77.5	12.4
All field-deployed ATSB	55.9	8.6	53.5	7.7	95.3	6.8	49.4	7.2	36.6	8.8	71.3	16.2	105.3	11.2	90.0	12.6	83.9	9.7

**Fig. 5:**
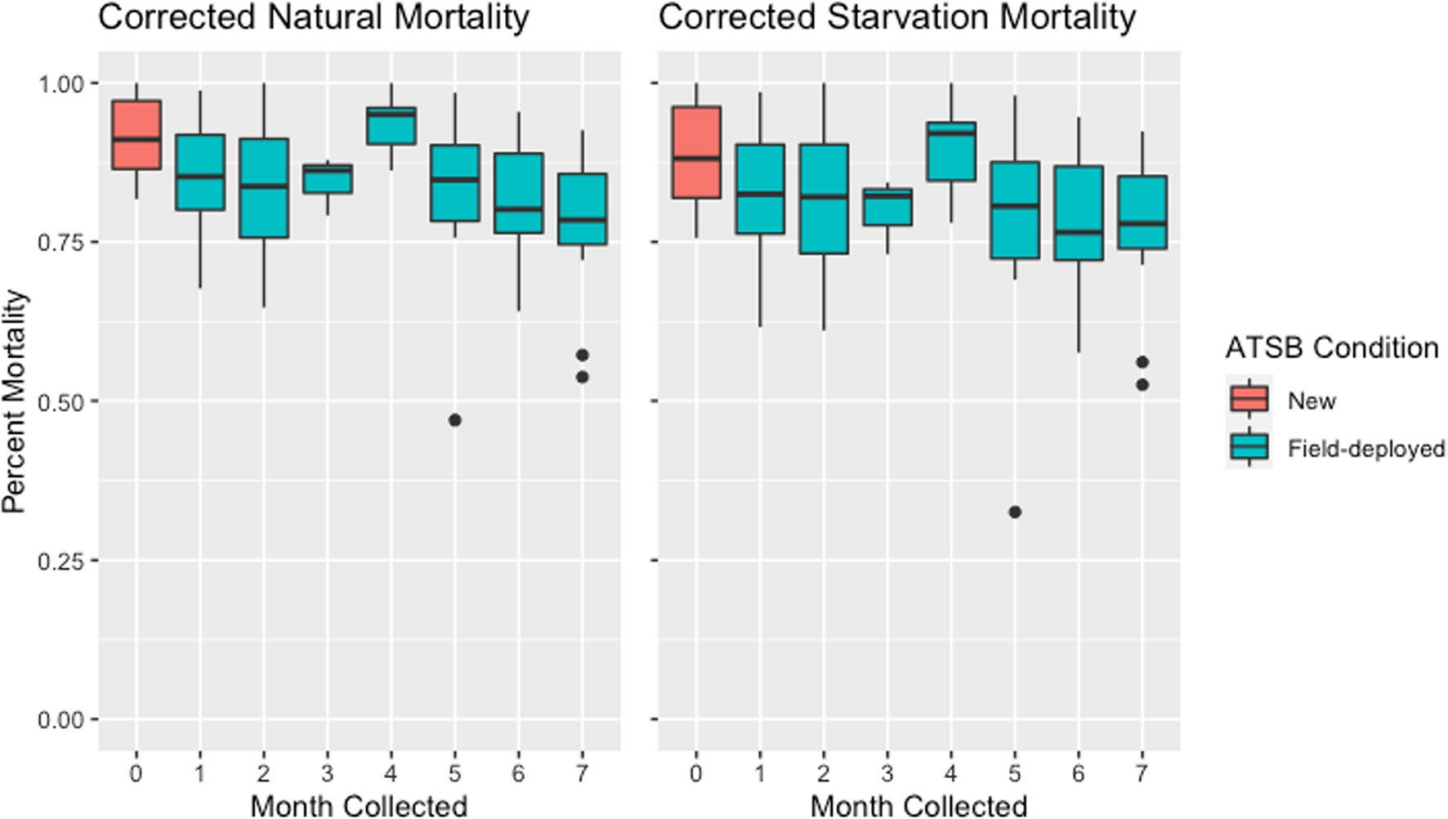
ATSB corrected mortality by collection round (left corrected for natural (sugar control mortality) and right panel corrected for starvation (water control) mortality)

Field-deployed ATSB stations had lower bioefficacy than non-deployed ATSB stations (83.9% vs. 91.4% corrected for natural mortality [95% CI for difference 3.0–12.2%, t = 3.37, p = 0.002]). 24-h mortality (54.7%) was significantly lower than 48-h mortality [95% CI for difference − 23.46% to − 33.33%, t = − 11.33, p < 0.001]. There was relatively little variation in corrected mortality between months for those ATSB stations that had been deployed to the field. The ATSB stations collected during sampling round four (after four months of deployment) showed significantly higher mortality than field-deployed ATSB stations collected during other rounds. Round four mortality was similar to but higher than ATSB stations that were non-deployed (2.2% higher than non-field deployed ATSB stations (p = 0.6). Overall, the mean mortality levels remained over 80% in bioassays of field deployed bait stations throughout the study period (Fig. 5).

## Results of unadjusted analysis of ATSB collection round on corrected mortality (bioefficacy).

There was a general downward trend in corrected mortality (~ 1.3%) per month of deployment in the entire dataset, but this trend was not statistically significant when non-deployed (month 0) ATSB stations were excluded from the analysis, indicating that there was no evidence of an overall trend in ATSB bioefficacy decline with duration of deployment (time) among the selected field deployed ATSB stations even when removing ATSB collected after approximately four months of deployment, as shown in Table 3.

**Table 3:** Effect of ATSB collection round on corrected mortality (bioefficacy)

Characteristic	Beta	95% CI^1^	p value
Condition			
New	–	–	
Field-deployed	− 0.08	− 0.14, − 0.01	0.027*
Month collected			
0	–	–	
1	− 0.07	− 0.15, 0.02	0.12
2	− 0.09	− 0.17, 0.00	0.045*
3	− 0.07	− 0.20, 0.06	0.3
4	0.02	− 0.06, 0.11	0.6
5	− 0.08	− 0.17, 0.00	0.046*
6	− 0.10	− 0.19, − 0.02	0.020*
7	− 0.14	− 0.22, − 0.05	0.002*
Round linear	− 0.01	− 0.02, 0.00	0.012*
Round linear (Field-deployed only)	− 0.01	− 0.02, 0.00	0.2
Round linear (Field-deployed only w/o round 4)	− 0.01	− 0.02, 0.00	0.2

Bold symbols reflecting indicates significance downward trend in the corrected mortality in those months round of the ATSB assessments.

^1^*CI* confidence interval

When disaggregated by sex, on average, male mosquitoes experienced 24% higher adjusted mortality (p < 0.001) than female mosquitoes on field deployed ATSBs. Female mortality declined slightly in field deployed ATSBs (1.5%, p = 0.2), while male mortality did not. However, the trend was non-significant whether month 4 was included or not (both models have the same Beta and p-value) (Table 2S). There was no evidence of interaction between the duration of deployment and sex.

## Discussion

This study aimed to assess the bioefficacy of ATSB stations deployed on structures in Western province, Zambia, for up to seven months. While *An. gambiae s.s.* mortality rates were lower for all field-deployed ATSB stations than for new non-deployed ATSB stations, the overall bioefficacy (summary of male and female 48-h mortality rates) of field-deployed ATSB stations remained high (greater than 80% when corrected for natural mortality) throughout the seven months of deployment. Of particular note, male *Anopheles* mosquitoes experienced a significantly higher average mortality rate (24% higher) than female *Anopheles* mosquitoes on field-deployed ATSB stations; female *Anopheles* mosquitoes experienced average mortality rates well below the 80% level, (71.3% overall mortality throughout the assessment period).

The observed differences between male and female mortality rates merit further attention in future studies given that only female mosquitoes are directly involved in malaria transmission. This result may represent more general biologic differences in male and female sugar feeding practices or could be due to specific differences between male and female *An. gambiae* Kisumu laboratory strain mosquitoes in feeding and mortality. Field testing of these bait stations reported by Chanda et al. [31] found that both male and female *An. gambiae* and *An. funestus* vector populations fed on attractive sugar bait stations (without insecticide) for the 3-month duration of the trial; however those studies could not assess the full extent of feeding characteristics or the effect on mortality (there was no toxicant used).

Retention of bioefficacy over the duration of the malaria transmission season is in accordance with findings from East and West Africa [23, 32], where other ATSB stations demonstrated high feeding rates and significant reductions in *An. gambiae s.l.* density and biting rates. Other studies [24] showed that high coverage with a combination of LLINs and ATSB methods for malaria vector control could result in substantial reductions in malaria transmission and are highly effective in arid environments regardless of competitive, highly attractive natural sugar sources in their outdoor environment [33]. This finding therefore supports the ATSB approach as a potential complementary tool that targets the sugar feeding behaviour of mosquitoes.

On opening Westham prototype ATSB stations from their packaging, leaching of small amounts of bait onto the membrane surface has been observed. It is possible that this contributes to bioassay mortality initially but that surface material is quickly detoxified or lost outdoors, accounting for the slight decrease in induced mortality observed. By the study design, ATSB stations collected during deployment were in good condition and did not have leaks, while a few had holes (< 3%). While the effects of bait station condition on bioefficacy were examined, dirtiness was the only condition found to significantly lower bioefficacy. As bait stations were selected based on their condition, there was low variation in the conditions of the stations tested, and, therefore, the study was not able to fully assess the impact of physical condition on bioefficacy.

Month four shows higher bioefficacy mortality, but this finding is not statistically significant when excluded from the analysis and only slightly increased the estimated bioefficacy of the ATSB stations over time when the analysis of inclusion and exclusion of that month was done. This is likely due to unexplained variation (most likely laboratory, insectary, and/or mosquito colony conditions), but the specific reasons for these ATSB stations showing anomalously high bioefficacy remain unexplained.

Other than a reduced bioefficacy consistent with having ever been deployed, no significant trend toward declining efficacy associated with collection month was identified. Importantly, the ATSB stations evaluated were selected for being in good condition and would not have been identified as needing replacement based on holes, leaks, mold. As such, these results show that this version of ATSB stations that remain in good condition retain bioefficacy in field conditions in Western Zambia over at least seven months provided that they have not become sufficiently degraded or damaged during deployment. The bioefficacy was only being assessed in terms of a laboratory cage mortality assay using laboratory colony of *An. gambiae s.s.* Kisumu strain. The feeding rate was not directly assessed, and the impact of time of exposure outdoors to the longer range attractiveness and competition with natural sugar sources was not tested in this study. Bioefficacy assessments on a random sample of ATSB stations irrespective of their current condition may be required to understand whether there is an association between ATSB station condition and mosquito mortality rates. Further research is also needed to determine the rate at which ATSB stations degrade with field deployment under varied conditions and to assess what types and effects of degradation in the field may result in changes to bioefficacy. More research is also needed to examine a diverse range of species (wild mosquitoes) to determine whether the observed pattern of mortality is consistent or if there are species-specific variations.

Although both male and female mosquitoes were included in the bioassays reported here, a focus on female mortality rates in future ATSB bioassays as standard measure of bioefficacy is critically important given that females are central to transmission and generally have lower mortality rates in controlled cage assays. Given the significant differences in 24-h and 48-h mortality rates, further experimentation with ATSB stations that include a tracer to identify feeding, such as uranine, may help to determine the optimum assessment period and quantify any delayed mortality rate following the feeding on the ATSB stations.

## Conclusions

Field-deployed ATSB stations retain bioefficacy for at least seven months provided they remain in good condition, however notable differences between male and female mosquitoes were observed with overall lower mortality observed among females. Initial deployment of ATSB stations appeared to reduce their bioefficacy by nearly 7.6% compared to stations that were tested immediately after removal from packaging. This might be due to exposure to outdoor environment and the differences in the timing of testing versus deployed ATSB stations. Despite this initial drop in bioefficacy, overall mortality levels for ATSB stations in good condition remained relatively high for the full seven months duration of the malaria transmission season in western Zambia.

### Supplementary Information

Supplementary Material 1.

## Data Availability

The annotated datasets and R code used to support the findings of this study are available from the authors upon reasonable request.

## Abbreviations

ATSB: Attractive targeted sugar bait (with insecticide)
WHO: World Health Organization
ITN: Insecticide-treated net
LLIN: Long-lasting insecticidal net
IRS: Indoor residual spraying
GTS: Global technical strategy for malaria
MoH: Ministry of health
NMEP: National malaria elimination programme
SOP: Standard operating procedure
MRT: Macha research trust
