# Supplementary material for: Residual bioefficacy of attractive targeted sugar bait stations targeting malaria vectors during seasonal deployment in Western Province of Zambia
Source: Malar J. 2024 May 29;23:169. doi: 10.1186/s12936-024-04990-3 (PMC11138038; doi:10.1186/s12936-024-04990-3)
Supplement: Supplementary file 1 — Supplementary Material 1. [file 12936_2024_4990_MOESM1_ESM.docx]

*Table S1: 24hr Sugar-fed Female Mosquito Mortality Per Trial*

| 24hr Sugar-Fed Female Mortality | |
| --- | --- |
| Trial | (%) |
| 0 | 0.00% |
| 1 | 2.86% |
| 2 | 1.79% |
| 3 | 4.08% |
| 4 | 0.00% |
| 5 | **11.43%** |
| 6 | 4.26% |
| 7 | 4.76% |

*Figure 1S: ATSB corrected mortality by collection round (left corrected for natural (sugar control mortality) and right panel corrected for starvation (water control) mortality) and time.*


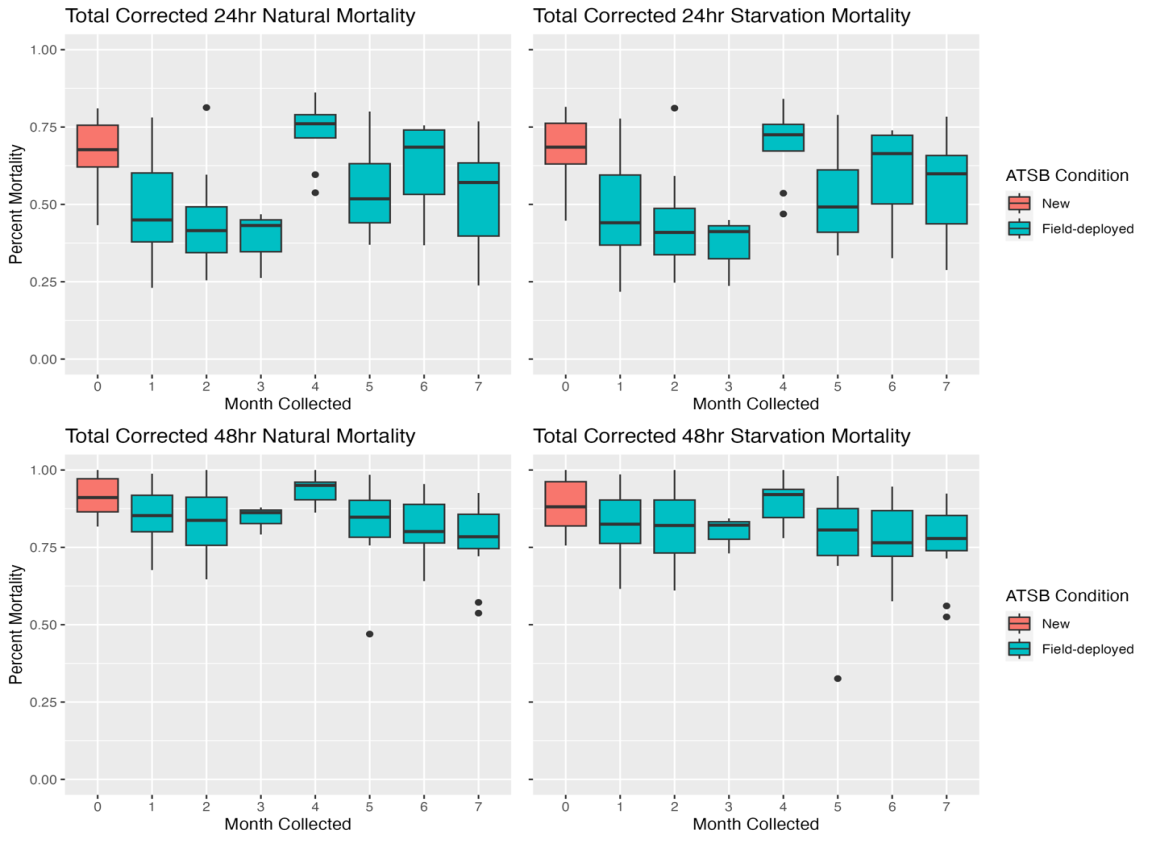


*Figure 2S: ATSB corrected mortality by collection round (left corrected for natural (sugar control mortality) and right panel corrected for starvation (water control) mortality), time, and sex.*


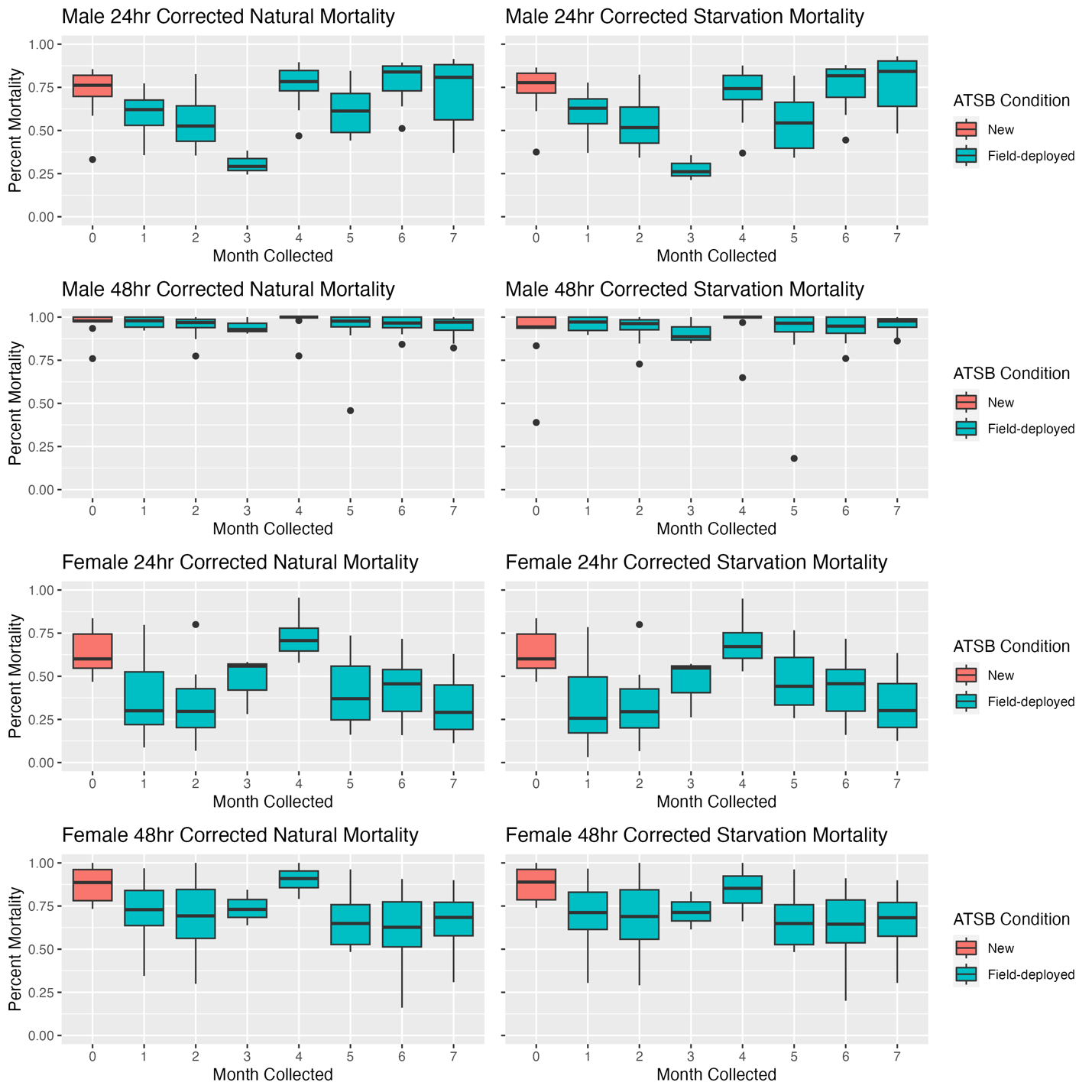


*Table 2S: Effect of ATSB collection round on corrected mortality (bioefficacy) by sex*

|  | **Total Mortality** | | | **Male Mortality** | | | **Female Mortality** | | | |
| --- | --- | --- | --- | --- | --- | --- | --- | --- | --- | --- |
| **Characteristic** | **Beta** | **95% CI**^1^ | **p-value** | **Beta** | **95% CI**^1^ | **p-value** | **Beta** | **95% CI**^1^ | **p-value** |  |
| **Condition** |  |  |  |  |  |  |  |  |  |  |
| **New** | — | — |  | — | — |  | — | — |  |  |
| **Field-deployed** | -0.08 | -0.14, -0.01 | **0.027** | -0.01 | -0.06, 0.04 | 0.6 | -0.16 | -0.27, -0.05 | **0.006** |  |
| **Month Collected** |  |  |  |  |  |  |  |  |  |  |
| **0** | — | — |  | — | — |  | — | — |  |  |
| **1** | -0.07 | -0.15, 0.02 | 0.12 | 0.01 | -0.06, 0.07 | 0.8 | -0.14 | -0.28, -0.01 | **0.042** |  |
| **2** | -0.09 | -0.17, 0.00 | **0.045** | -0.02 | -0.08, 0.05 | 0.6 | -0.19 | -0.32, -0.05 | **0.008** |  |
| **3** | -0.07 | -0.20, 0.06 | 0.3 | -0.02 | -0.12, 0.08 | 0.7 | -0.13 | -0.35, 0.08 | 0.2 |  |
| **4** | 0.02 | -0.06, 0.11 | 0.6 | 0.01 | -0.05, 0.08 | 0.7 | 0.03 | -0.10, 0.17 | 0.6 |  |
| **5** | -0.08 | -0.17, 0.00 | **0.046** | -0.04 | -0.10, 0.03 | 0.2 | -0.20 | -0.33, -0.06 | **0.005** |  |
| **6** | -0.10 | -0.19, -0.02 | **0.020** | -0.01 | -0.07, 0.06 | 0.8 | -0.25 | -0.39, -0.12 | **<0.001** |  |
| **7** | -0.14 | -0.22, -0.05 | **0.002** | -0.02 | -0.09, 0.05 | 0.6 | -0.22 | -0.36, -0.08 | **0.002** |  |
| **Round Linear** | -0.01 | -0.02, 0.00 | **0.012** | 0.00 | -0.01, 0.00 | 0.4 | -0.02 | -0.04, -0.01 | **0.006** |  |
| **Round Linear (Field-deployed only)** | -0.01 | -0.02, 0.00 | 0.2 | 0.00 | -0.01, 0.01 | 0.5 | -0.01 | -0.04, 0.01 | 0.2 |  |
| **Round Linear (Field-deployed only w/o round 4)** | -0.01 | -0.02, 0.00 | 0.2 | 0.00 | -0.01, 0.01 | 0.5 | -0.01 | -0.03, 0.01 | 0.2 |  |
| ^1^CI = Confidence Interval | | | | | | | | | | |
